# 

**Published:** 1916-10-14

**Authors:** 


Dr. T. W. Jones, the borough medical officer of Wrexham, having been granted leave to serve with the Forces, the Corporation has arranged to appoint Dr. Thomas Roberts, the county medical officer, as temporary medical officer for the borough and rural district of Wrexham, at a joint salary of £100 per annum; and Dr. H. Drinkwater temporary medical superintendent of the Joint Fever Hospital, at a salary of £75. Dr. T. W. Jones is being granted an allowance sufficient to bring his military pay up to the standard of his civic remuneration.

### Teaching at the Royal Sanitary Institute.

The autumn session of lectures and demonstrations arranged by the Royal Sanitary Institute includes courses of instruction in some of the new fields of public-health work.

The course for "women health visitors, tuberculosis visitors, school nurses, school teachers, and other students" covers a very wide range of subjects, but must, including, as it does, single lectures on water, elementary statistics, and house drainage, be often introductory; especially perhaps in the case of the one lecture devoted to first-aid, treatment of injuries, ailments, and accidents—and including, *inter alia*, bleeding, "fits," drowning, and preparation for operation! Candidates for the examination for women health visitors and school nurses must produce evidence of nursing training: this is a wise provision. The certificate of the Institute is named by the Local Government Board as one of the qualifications for appointment as health visitor or school nurse.

Supplementary to this course for health visitors, etc., is one for maternity and child-welfare workers, open to women students only. The syllabus of the ten lectures is well arranged, and the lectures are to be supplemented by demonstrations at the Parkes Museum. The first examination for maternity and child-welfare workers is to be held in London on December 8 and 9 next. It is to consist of written papers, *vivâ-voce* questions, and practical work; the fee is £3 3s. The scheme is of hopeful promise, and should turn out thoroughly equipped women workers for this new and important branch of public assistance.

RATES OF SUBSCRIPTION: Twelve months. 6/6; Six months, 4/-; Three months, 2/-; post free to any address in the United Kingdom. Abroad, Twelve months, 8/8; Six months, 5/-; Three months, 2/6, post free. THE HOSPITAL "Index" to Volume LX., April to September 1916, is now ready, price 1½d. per copy, post free. Address The Manager, "THE HOSPITAL," The Hospital Building, 28/29 Southampton Street, Strand, London, W.C.

